# En bloc kidney transplant from an 18-month-old donor to an adult recipient: Case report and literature review^☆^

**DOI:** 10.1016/j.ijscr.2013.08.006

**Published:** 2013-08-28

**Authors:** B. Patrice Mwipatayi, Chee Weng Leong, Pradeep Subramanian, Alarick Picardo

**Affiliations:** a University of Western Australia, School of Surgery, Crawley WA 6009, Australia; b Department of Vascular Surgery, Royal Perth Hospital, Level 2, MRF Building, Perth, WA 6001, Australia

**Keywords:** Paediatric kidney donor, En bloc kidney transplant, Single kidney transplantation

## Abstract

**INTRODUCTION:**

There is an ever-increasing need for organ donations globally. Paediatric kidney transplantation into adult recipients is a well-recognised technique to expand the donor pool. The transplantation can be done either via en bloc kidney transplant (EBKT) or as single kidney transplantation (SKT).

**PRESENTATION OF CASE:**

An EKBT from a 18-month-old (15 kg) male patient was transplanted in a 35-year old, 85 kg male with end stage renal failure (ESRF), secondary to Focal Segmental Glomerulosclerosis (FSGS) on haemodialysis. Post-operative recovery was uneventful. Immuno-suppressant drugs used were tacrolimus, basiliximab and prednisolone. Doppler ultrasound scans performed post-operatively showed normal renal resistive indices in both kidneys. Serum creatinine decreased from 1200 to 170 μmol/L 57 with eGFR improving from 4 to 38 mL/min/1.73 m^2^ at four weeks post-transplant.

**DISCUSSION:**

Given the low incidence of paediatric donors, EBKTs are relatively uncommon and subsequently published series tend to be centre specific with small numbers. The graft survival rates tell us that paediatric kidney donors should not be considered as marginal transplants. The difficulty is in determining when it is more appropriate to perform a paediatric EBKT as opposed to splitting and performing two SKT. Unfortunately there are no widely accepted guidelines to direct clinicians.

**CONCLUSION:**

This case report highlights the first EKBT performed at our institution. The current literature demonstrates that paediatric donors are excellent resources that should be procured whenever available.

## 1 Introduction

There is an ever-increasing need for organ donations globally. In 2011, a total of 1099 organs were transplanted from 354 donors, the highest annual amount to date. 606 of these were renal transplants, 129 of which were performed following cardiac death. Despite this, there are still a great number of patients on the waitlist for a renal transplant.

Paediatric kidney transplantation into adult recipients is a well-recognised technique to expand the donor pool. There are however, conflicting opinions regarding en bloc kidney transplantation (EBKT) versus single kidney transplantation (SKT).

We report our institution's first successful EBKT from a small, paediatric, deceased donor into an adult recipient and summarise the current literature.

## 2 Case report

The Donor (Maastricht category III) was an 18-month-old, 15 kg male patient (BSA: 1.1 m^2^) who died of drowning. Only his kidneys were accepted for transplantation for a single recipient. The recipient was a 35-year old, 85 kg male (BSA: 2.05 m^2^) with end stage renal failure, secondary to Focal Segmental Glomerulosclerosis (FSGS) on haemodialysis. The donor-recipient antigen mismatch was 3/6.

**Fig. 1:**
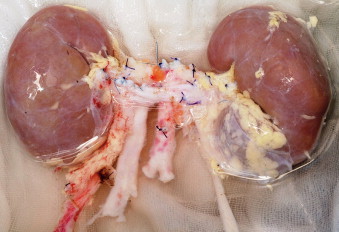
Prepared kidneys retrieved en bloc with the proximal end of the aorta and inferior vena cava oversewn and ureters sectioned as close to the bladder (a) and (b).

**Fig. 2:**
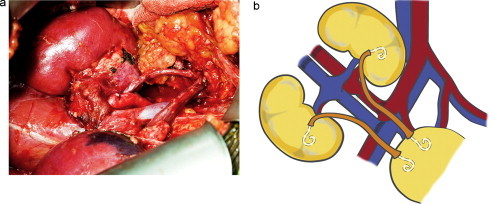
Transplantation of the kidney. The proximal aortic segment was anastomosed end-to-side to the right external iliac artery (EIA). The IVC (inferior vena cava) was sutured end-to-side to the right external iliac vein (EIV). The two ureters were sutured separately to the dome of the urinary bladder with two 6F, 10 cm stents in place (a) and (b).

The kidneys were retrieved en bloc with the proximal end of the aorta and vena cava oversewn and ureters sectioned as close to the bladder as possible (Fig. 1). The distal ends of the aorta and vena cava was anastomosed end-to-side to the recipient external iliac artery and vein, respectively, with 6–0 prolene. The ureters were implanted separately, to avoid stenosis. Double-J catheters (6-French; 14 cm) were implanted in each ureter and were withdrawn at one-month post transplantation. The spaced cystoureteric anastomoses (according to Lich-Gregoir technique) were performed using 3–0 PDS as shown in Fig. 2(a) and (b). Three thousand units of intravenous heparin were administered during the procedure.

The cold and warm ischaemic times were 405 and 45 min, respectively.

**Fig. 3:**
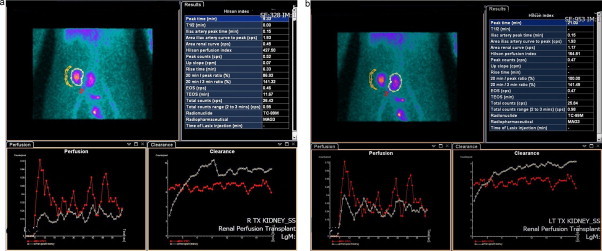
Renal perfusion study – MAG 3 (a) and (b).

Post-operative recovery was uneventful. Immuno-suppressant drugs used were tacrolimus, basiliximab and prednisolone. Doppler ultrasound scans performed day one, three, 14 and 21 post-operatively showed normal renal resistive indices in both kidneys (0.59–0.70). Serum creatinine decreased from 1200 to 170 μmol/L with eGFR improving from 4 to 38 mL/min/1.73 m^2^ at four weeks post-transplant. A CT scan performed on day one showed that the kidneys were not malrotated and in satisfactory position. A renal perfusion study, using a Tc-99m MAG.3 performed 24 h after transplantation, demonstrate that the renogram curve shows a rising pattern bilaterally with similar count activity, implying fairly symmetrical renal function. Excreted tracer is seen in the bladder from 6 min post-injection. Activity peaks on the kidney located to the right side at 21 min post injection and plateaus from 9 min post injection on the kidney located to the left side (Fig. 3(a) and (b)). But quantitative parameters should be interpreted with caution in this patient due to the small size of the allograft kidneys relatively to the feeder vessels and overlay of the left kidney over the right iliac artery.

## 3 Discussion

The decision of how to best utilise donor paediatric kidneys is difficult. Historically, the main factors that fostered reluctance in using paediatric kidneys included technical difficulties in salvage and transplantation, early graft failure, high rates of graft thrombosis, concern for hyper-filtration injury, frequent rejection episodes, suboptimal nephron mass and a lack of long term graft and survival outcomes.^1^

EBKT was originally developed to increase the transplanted nephron mass and to overcome the technical challenges of small calibre vessels in paediatric donors. While it has made the technical aspects of procuring and transplanting small paediatric kidneys easier, challenges are still present and surgical experience and technique has been shown to greatly affect outcomes.^2^ First described in a xenograft model in 1908, many refinements and adaptations of the en bloc method have been described to reduce complications.^3,4^

Given the low incidence of paediatric donors, EBKTs are relatively uncommon and subsequently published series tend to be centre specific with small numbers. Several authors have reported outcomes using the United Network of Organ Sharing (UNOS) or Scientific Registry of Transplant Recipients (SRTR) registries.

Bhayana et al. used UNOS data (1988–2006) and reported that paediatric EBKT has better longer term graft survival (GS) than paediatric single kidney transplant (SKT) and the best long-term GS over adult standard criteria donor transplant (SCDT) despite higher graft loss during the first 12 months post-transplant.^4^ Graft survival (GS) probability estimates at 5 and 10 years were 74.8% and 64.0%, respectively, for EBKT compared to SKT rates of 65.2% and 52.5%, and SCDT rates of 75.2% and 57.4%, respectively (*P* < 0.001).

Thomusch et al. recently reported a 20-year graft survival and function analysis from a matched pair study between paediatric EBKT and cadaveric adult donor grafts. With a mean EBKT donor age of 15 months he also reported a higher early graft loss (first post-operative year) but superior long-term outcomes in graft survival and function with EBKT (1, 5, 10-year GS of 83.1%, 76%, 73.9% vs. 89.6%, 78.7%, 57.8%, respectively).

These graft survival rates tell us that paediatric kidney donors should not be considered as marginal transplants. The difficulty, however, is now in determining when it is more appropriate to perform a paediatric EBKT as opposed to splitting and performing two SKT. Unfortunately there are no widely accepted guidelines to direct clinicians but instead, based on earlier registry analysis <5 years or <20 kg is being crudely used as the cut-off where EBKT is preferential to SKT.

More recently, however, Mohanka et al. compared SKT and EBKT from paediatric donors’ ≤15 kg and no significant difference was noted in one-year survival rates between EBKT (79%) and SKT (86%).^5^

Similarly, using the SRTR data (1995–2007), Kayler et al. showed that graft survival of ideal standard criteria donors was similar to SKT from donors weighing ≥35 kg and EBKT from donor's ≥10 kg. Between donor weights of 10–34 kg, EBKT had superior outcomes over SCDT. However, the protective benefit was less than half as compared to SKT and so authors concluded that from a resource perspective, splitting these kidneys would increase overall total graft years to the recipient population.^6^

Laurence et al. came to a similar conclusion through the creation of a decision analysis model to predict life years gained for patients with ESKD on the transplant waiting list depending on whether they received EBKT or SKT.^7^ A greater overall life expectancy gain was achieved using SKT because two recipients were yielded per donor, this more than compensated for the increased risk of graft failure associated with SKT.

Graft failure is, however, a major concern for all paediatric donors (EBKT or SKT), with most single centre studies and transplant registries reporting early graft failure at higher rates than those of standard adult donors. After approximately 12 months post-transplant, survival outcomes with EBKT equal that of SCDT, with one study even showing superiority of EBKT over living donor kidneys.^4,8^ It is, therefore, crucial to identify and minimise risk factors for graft failure during this early post-transplant period.

The most common causes for early graft failure are vascular complications, with reported rates of vascular thrombosis between 2.5 to 12.5% in small paediatric donors, considerably higher than thrombosis rates for standard adult donors (∼1.8%).^4–6,8^ Surgical technique, peri-operative blood pressure management, vessel calibre, vessel or kidney torsion, hypercoaguable states, haematomas, lymphocytes and acute rejection have all been suggested as causes for thrombosis.^5,9^ Risk factors for thrombosis in all renal transplants include young donor age (<5 years), cold ischaemia time >24 h, previous recipient transplantation, African American race and increased panel reactive antibody.^5,10,11^ In paediatric donors, the absence of an aortic patch during SKT and donor age less than 12 months with EBKT are also risk factors for graft thrombosis.^8,11^ Routine anticoagulation has not been shown to affect graft thrombosis rates.^2,12^

Other causes for graft failure include acute or chronic rejection, medication non-compliance, primary non-function, transplant pyelonephritis, graft dislocation and surgical complications.^13,14^ Using the SRTR data, Pelletier et al. reported the recipient and donor characteristics that independently predicted graft failure with small paediatric donor kidneys being recipient age >65, African American or Asian recipient race, low donor weight and diabetes as the cause of ESRD.^15^

There have also been concerns about donor recipient weight disparity especially in paediatric to adult transplants. Consequently, kidneys from small paediatric donors have often been primarily transplanted into other children or small adults as a way of matching nephron mass and ensuring functional requirement of recipients are met. The rationale behind this regarded concerns about causing hyperfiltration injury, whereby compensatory changes in the transplant kidney result in hypertension, proteinuria, and glomerulosclerosis and, ultimately, graft failure.^16^ This has been shown to occur in adult cohorts with large recipients receiving kidneys from small donors (based on body surface area) having a 43% increased risk of late graft failure compared with medium recipients receiving kidneys from medium donors.^17^ However, these concerns of suboptimal nephron mass in very young donors have largely been discredited. Numerous studies have demonstrated that paediatric transplanted kidneys underwent compensatory hypertrophy to reach normal adult size by approximately 18 months and thereby actually improve in function over time and maintain better glomerular filtration than adult transplanted kidneys.^18,19^ Bhayana et al. reported that with paediatric SKT, the eGFR while initially lower, equalised SCD at 6 months, became higher at 12 months and continued to increase until 36 months. As such, 50mths post-transplant eGFR was significantly higher in SKT vs. SCD (57.4 vs. 47.9 ml/min/1.73 m^2^, *P* < 0.0001).^4^ Recently Kayler et al. confirmed that increasing recipient BMI was not a clear risk factor for outcomes or graft function with small paediatric donors.^16^

Acute rejection is another concern with paediatric kidney donors having high rates of acute rejection compared to standard adult donors.4 Bhayana reported acute rejection rates of 9% in SKT compared to 6% with EBK and similar results have been reported in other series.^20–22^ The role of induction strategies is unclear. Some agents (basiliximab, Thymoglobulin, IL-2 receptor antagonists) have not been shown to significantly reduce rejection rates, however, other observations are that induction immunosupression can have benefit.^6,12,22,23^ Urinary complications in small paediatric donor kidneys have been reported between 2.5 and 11% with no significant differences between EBKT and SKT.^5^

## 4 Conclusion

In conclusion, the current literature demonstrates that paediatric donors are excellent resources that should be procured whenever available. The decision to perform EBKT or splitting to perform two SKT is difficult. While some centres have reported excellent outcomes with SKT, overall graft survival is inferior to EBKT, especially with lower donor weights with greater technical difficulty. In our institution and throughout Australia, as paediatric donors are uncommon and surgical experiences limited, performing EBKT may be prudent, especially in young paediatric donors.

## Conflict of interest

We, the authors declare that there is no conflict of interest.

## Funding

None.

## Consent

Authors confirm that written informed consent was obtained from the patient for publication of this case report and accompanying images. A copy of the written consent is available for review by the Editor-in-Chief of this journal on request.

## Author contributions

B. Patrice Mwipatayi: study design, data collections, writing and final approval.

David Leong Chee Weng: data collection, writing.

Pradeep Subramanian: data collection, writing.

Alarick Picardo: writing, editing.
